# A Rare Congenital Perineal Groove

**DOI:** 10.1155/2022/8797205

**Published:** 2022-10-27

**Authors:** Enyew Abate

**Affiliations:** Department of Obstetrics and Gynecology, College of Medicine and Health Sciences, Bahir dar University, Ethiopia

## Abstract

**Background:**

Perineal groove is a rare congenital anomaly characterized by nonepithelialized mucous membrane that appears as an erythematous sulcus in the perineal midline, extending from the posterior vaginal fourchette to the anterior anal orifice. It is one of the uncommon anomalies of anogenital region that is unknown to many clinicians, and it is usually misdiagnosed as an anal fissure, perineal trauma, diaper dermatitis, infection, or sexual abuse. *Case Presentation*. Seven-day-old female neonate was brought by her parents after they observe reddish discoloration of her genitalia. Upon examination, there was a vertical perineal wet groove-like unkeratinized erythematous lesion with no bleeding, sign of infection, or inflammation that extends from the posterior vaginal fourchette to the anal rim. The newborn was sent home after counseling with a reassessment plan in case of complications.

**Conclusions:**

Recognition of the congenital perineal groove at birth is important for the health care providers to deliver an appropriate parental counseling and appropriate follow-up.

## 1. Background

Perineal groove is a rare congenital anomaly characterized by nonepithelialized mucous membrane that appears as an erythematous sulcus in the perineal midline, extending from the posterior vaginal fourchette to the anterior anal orifice [1, 2]. It is a benign rare entity first described by Stephens in 1968 [3]. It is unknown to many clinicians, and it is usually misdiagnosed as an anal fissure, perineal trauma, diaper dermatitis, infection, or sexual abuse [4]. The diagnosis of the defect is set mainly on the clinical assessment of the lesion [5]. This is the first case reported in Ethiopia.

## 2. Case Presentation

A seven-day-old female newborn from Bahir Dar city was brought to Afilas General Hospital by her parents with the complaint of reddish discoloration of the skin around the genitalia (like fresh wound). The neonate was born to a 30-year-old Para II mother through spontaneous vaginal delivery with no instrumentation at gestational age of 39 + 6 weeks with birth weight of 3400 grams and Apgar score of 8 and 10 in the first and fifth minutes, respectively. The total duration of labor was 10 hours and rupture of membrane was intrapartum. The mother had regular antenatal care follow-up and it was uneventful. The mother has no history of cigarette smoking, alcohol consumption, or substance abuse. Both the father and mother have no history of congenital birth defects in the family. There was no history of local trauma, defecation issues, and urinary problem with the newborn.

**Figure 1:**
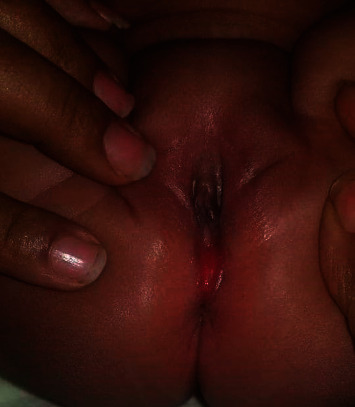
Physical examination finding showing the perineal groove.

Physical examination of the neonate was normal in other body systems. On genital examination, there was around 1.5 cm wet groove-like erythematous vertical lesion which extends from the posterior vaginal fourchette at 6 o'clock to the anal rim (Figure 1). The lesion has no bleeding, no sign of infection, or inflammation. Urethra and vaginal orifices were intact and located at normal position. The anus is perforated with normal anorectal examination with neonate passing meconium and no fistula. Ultrasound of the kidneys and pelvis was normal. The examination revealed a congenital perineal groove, and the parents were counseled about expectant management and sent home with appointment to undergo follow-up examinations.

## 3. Discussion

Perineal groove is generally a wet sulcus lesion extending from the posterior vaginal fourchette to the anterior anus [4]. It is mostly presented in female infants as an isolated anomaly but can be found in males as well [6]. Incidence of this congenital anomaly is underestimated as it may be unrecognized earlier at birth and/or later misdiagnosed as a diaper rash, contact dermatitis, or trauma [1]. Our case is female with no associated anomaly which was not diagnosed at birth.

It is a congenital malformation that consists of three main features: (1) a wet groove in the midperineum between the fourchette and the anus, (2) normal formation of the vestibule including the urethra and vagina, and (3) hypertrophy of the minoral tails that skirt the perineum and course posterior to join at the anus or surround it [7].

There are several controversial embryological theories regarding the formation of perineal groove [2, 4]. This malformation may be a result of failure of fusion of the median genital folds, which are located on the midline [4]. The second theory states that perineal groove and perineal canal occur as uroanal septal defects between the 5th and 8th weeks of gestation after the normal urorectal septum forms [2, 4]. Abnormal expression of some genes like bone morphogenic protein 4 (BMP4) may be associated with perineal groove [4]. A third theory, justifying this defect in both sexes, is the failure of the development of external genitalia during the fusion of labioscrotal folds with ectoderm forming the midline raphe. When part of this merging does not take place, the raphe is replaced by the groove [2]. Even if in majority of cases the defect is presented as isolated defect, there is case report happening in monozygotic twins [8].

Perineal groove could be complete or incomplete. The complete perineal groove extends from the vaginal fourchette to the anus, while the incomplete type either extends from the vaginal fourchette to the middle of the perineum or from the anus to the middle of the perineum [9].

Few cases have been published in medical literatures [5, 10]. Definitive diagnosis is based on clinical examination, and occasionally, skin biopsy may be performed with limited importance [7]. This malformation is usually misdiagnosed sexual abuse, anal fissure, erosive diaper rash, diaper dermatitis, or ulcer [1, 2, 4].

Perineal groove usually resolves spontaneously, and complete epithelialization occurs by the age of 1 to 2 years [1, 7]. Surgical correction can be for cosmetic reason, if the lesion is not epithelialized by the age of 2 years and in patients with repeated inflammation or infection of the nonepithelized area [1, 11].

## 4. Conclusions

Correct recognition is important to avoid misdiagnosis and unnecessary investigations. I report this case to raise awareness and for doctors to provide appropriate advice and follow-up recommendations for parents of affected infants.

## Data Availability

All data are included in the manuscript.

## References

[1] Harsono M., Pourcyrous M. (2016). Perineal groove: a rare congenital midline defect of perineum. *American Journal of Perinatology Reports*.

[2] Boutsikou T., Mougiou V., Sokou R. (2019). Four cases of perineal groove—experience of a Greek maternity hospital. *Medicina*.

[3] Stephens F. D. (1968). The female anus, perineum and vestibule: embryogenesis and deformities. *Australian and New Zealand Journal of Obstetrics and Gynaecology*.

[4] Cheng H., Wang Z., Zhao Q., Zhu H., Xu T. (2018). Perineal groove: report of two cases and review of the literature. *Frontiers in Pediatrics*.

[5] Ajaj O. A. (2020). Perineal groove: a rare perineal malformation. *Asian Research Journal of Gynaecology and Obstetrics.*.

[6] Sekaran P., Shawis R. (2009). Perineal groove: a rare congenital abnormality of failure of fusion of the perineal raphe and discussion of its embryological origin. *Clinical Anatomy*.

[7] Garcia-Palacios M., Mendez-Gallart R., Cortizo-Vazquez J., Rodriguez-Barca P., Estevez-Martinez E., Bautista-Casasnovas A. (2017). Perineal groove in female infants: a case series and literature review. *Pediatric Dermatology*.

[8] Harsono M., Yanishevski D., Pourcyrous M. (2021). Congenital perineal groove defect in monozygotic twin infants: a literature review. *American Journal of Perinatology Reports.*.

[9] Naji H., Ali H. R. (2021). Perineal groove: is it more common than we think? Clinical characteristics of four cases and review of literature. *Pediatric Reports.*.

[10] Samuk I., Amerstorfer E. E., Fanjul M. (2020). Perineal groove: an anorectal malformation network, consortium study. *The Journal of pediatrics*.

[11] Wojciechowski M., Van Mechelen K., Van Laere D. (2019). Congenital perineal groove. *Archives of Disease in Childhood*.

